# The Use of Silicone Vaginal Repair Models as an Adjunct to Mannequins for Simulation Training in Sexual Assault Clinical Learning for Obstetrics and Gynecology Medical Residents

**DOI:** 10.7759/cureus.7410

**Published:** 2020-03-25

**Authors:** Megan Comeau, Christine Goudie, Deanna Murphy, Erika Fowler, Adam Dubrowski

**Affiliations:** 1 Medicine, Faculty of Medicine, Memorial University of Newfoundland, St. John's, CAN; 2 Medical Education and Simulation, Memorial University of Newfoundland, St. John's, CAN; 3 Obstetrics and Gynecology, Memorial University of Newfoundland, St. John's, CAN; 4 Health Sciences, Ontario Tech University, Oshawa, CAN

**Keywords:** simulation, obstetrics and gynecology, sexual assault, perineal repair, medical education, 3d printing, clinical learning

## Abstract

Within urgent care scenarios in obstetrics and gynecology, there is little educational development surrounding sexual assault simulation scenarios, which reveals a need for increased rehearsal opportunities within medical education. Simulation-based medical education using mannequins, standardized patients, and anatomical silicone models have been suggested as a means to improve such parameters by providing realistic training for residents in the rehearsal of sexual assault scenarios and the application of forensic evidence kits. However, sexual assault training is often only provided to emergency medicine physicians and clinical staff and is not currently a mandatory component of obstetrics and gynecology residency programs across most national academic institutions.

This technical report describes the development, implementation, and user-based evaluation of a simulation exercise within a sexual assault clinical learning scenario that uses a silicone vaginal model produced using a three-dimensional (3D) printed mold. The silicone model was covered in artificial blood and attached to an existing mannequin to simulate an examination following a violent sexual assault, which resulted in vaginal lacerations. The clinical room in which the simulation was held reflected that of an operating room in an emergency department, complete with simulation confederates acting as an attending general surgeon, nurse, and anesthesiologist. The obstetrics and gynecology residents acted as the urgent care providers being called into the operating room. The residents were briefed by the general surgeon upon arrival and scrubbed into the surgery. Next, they examined and repaired vaginal lacerations on the silicone model attached to the simulation mannequin. Finally, the residents followed up the clinical simulation with an opportunity to rehearse patient communication and empathy by consoling a standardized patient who acted as the victim of the sexual assault.

The purpose of this technical report is to evaluate the efficacy of a silicone vaginal model created from a 3D printed mold, which included a second-degree laceration, to train obstetrics and gynecology residents in the repair of injuries resulting from sexual assault. An evaluation survey was completed by attending residents and the results were strongly in favor of using such anatomical silicone models to increase realism and for the improvement of procedural competency in repairing vaginal lacerations caused by sexual assault.

## Introduction

Three-dimensional (3D) printing within simulation-based medical education (SBME) is becoming increasingly accessible and prominent in medical education due to a need for tactile and haptic learning prior to bedside application [1]. SBME learning provides trainees with opportunities to develop and improve their technical skills prior to contact with patients, which better prepares them to perform surgical procedures in the operating room [2]. Moreover, with increased emphasis placed upon patient safety, SBME training allows residents to perfect these skills in a safe, clinical environment where they have the freedom to make errors and receive immediate instructor feedback [3]. SBME often utilizes both standardized patients and standardized confederates. A standardized patient is an actor who has been specially trained to consistently portray a particular role, which helps ensure consistency in the training of medical learners [4]. A standardized confederate is a trained medical professional who guides the scenario and is able to provide instantaneous feedback [5].

In recent years, SBME has been used to enhance medical training in a variety of specialties such as cardiology, neurosurgery, and nursing, with positive learning outcomes [6-8]. In fact, SBME is considered a superior learning tool as compared to traditional didactic teaching methods for polishing technical surgical confidence and competency [9]. Despite success in adjunct areas, it appears that SBME has been integrated at a slower rate within obstetrics and gynecology (OB/GYN) residency programs, due mostly to the lack of affordable and effective task trainers [3]. With the rise and accessibility of 3D printing, OB/GYN has begun to see the development of innovative, low-cost simulation models, such as cervical models used to train residents in detecting cervical cancers, providing residents with improved learning opportunities [10]. Affordable silicone models have also been developed to assist with learning the repair of postpartum perineal lacerations, especially those involving the anal sphincter [11]. Since many possible scenarios may not naturally present themselves throughout residency training, SBME is a method to ensure that clinical trainees become proficient in specific skills.

Within urgent care scenarios in OB/GYN, there is little educational development surrounding sexual assault simulations, which reveals a need for increased rehearsal opportunities within medical education. Appropriate and compassionate management is imperative when providing care to sexual assault patients. Primarily, it is essential that physicians communicate effectively with victims of sexual assault. Physicians should ensure the patient’s confidentiality and safety and acknowledge the patient’s bravery in disclosing such sensitive information. Furthermore, physicians must accept this information in a non-judgemental way and validate the patient’s response to the sexual assault. Beyond effective communication, physicians must determine whether the patient would like to take legal action. If the patient wishes to take legal action, the physician ought to make a timely referral to a sexual assault nurse examiner (SANE), who will collect forensic evidence. If the patient is unsure about their decision to take legal action, forensic evidence may still be collected in the event that the patient later decides to take legal action. It is important that the physician takes detailed notes of the encounter should they be required for evidence. Appropriate management of sexual assault victims also includes providing emergency contraception if required. The physician should also perform a detailed general and genital exam, to assess the injuries sustained from the assault. If no injuries were sustained, the physician should reassure the patient. If the physical exam reveals injury, the physician must manage those appropriately. Finally, the physician must assess the patient’s risk for pregnancy and sexually transmitted infections [12].

It is also important for residents to understand the various clinical roles involved in sexual assault scenarios, including SANEs which have become more widespread in the treatment of urgent care victims [13]. SANE nurses are specifically trained to deal with sexual assault cases, including the application of forensic evidence kits, and it is thought that their involvement in patient care has resulted in improved outcomes [13]. In addition, their involvement has been a driving force in the increased rates of prosecution and conviction of perpetrators in sexual assault cases [14]. Unfortunately, SANE programs are not available at all hospitals and are disproportionately inaccessible in rural communities [13]. Although an important role in the treatment of sexual assault victims, SANE programs often decrease a resident’s exposure to such cases, resulting in decreased confidence and competency-related skills, especially regarding the use and application of forensic evidence kits [14]. SBME has been suggested as a means to improve such parameters, by providing realistic training for residents in the rehearsal of such assault cases. However, such training is often only provided to emergency medicine physicians and clinical staff and is not a mandatory or standardized component of the OB/GYN residency program across most national academic institutions [3,13].

The purpose of this technical report is to present the user-based product evaluation of a silicone vaginal model created from a 3D printed mold, which was used in conjunction with an existing simulation mannequin. The vaginal model included a second-degree laceration to train residents in the repair of injuries resulting from sexual assault. OB/GYN residents had an opportunity to rehearse their surgical skills in a sexual assault simulation scenario in a safe clinical environment using a combination of simulation resources such as a mannequin, attached silicone vaginal model with lacerations, standardized medical staff, and a standardized patient. Such hybrid simulation scenarios are thought to be superior as compared to traditional simulation methods, whereas it provides advanced sensory training involving haptic feedback, auditory response, and visual cues [15]. Such specific training is considered to be a rarity in OB/GYN residencies, however, it is considered effective in providing residents with increased awareness and advanced hands-on procedural learning through the presentation of an urgent care sexual assault scenario.

## Technical report

**Figure 1:**
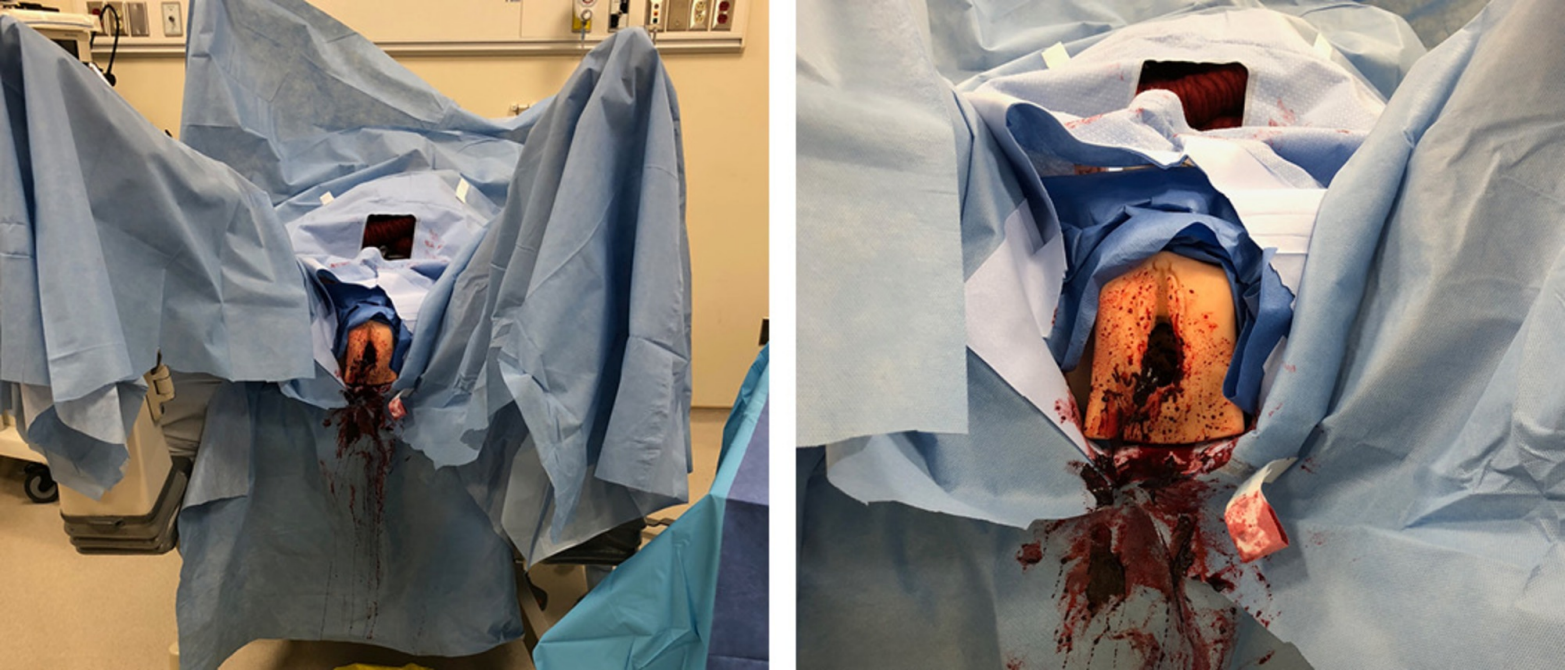
Sexual assault scenario using a silicone model to simulate injuries incurred by the patient

In previous studies, the same silicone models were used for the rehearsal of first and second-degree postpartum laceration repair within SBME at Memorial University of Newfoundland (MUN) [11]. The silicone model in this scenario was repurposed and attached to a simulation mannequin within a clinical learning environment. The model was then covered in artificial blood to simulate the female anatomy post-assault (Figure 1).

The room that the simulation scenario was hosted in reflected that of an operating room in an emergency department, complete with simulation confederates acting as an attending general surgeon, nurse, and anesthesiologist, with the OB/GYN residents acting as the urgent care providers.

**Figure 2:**
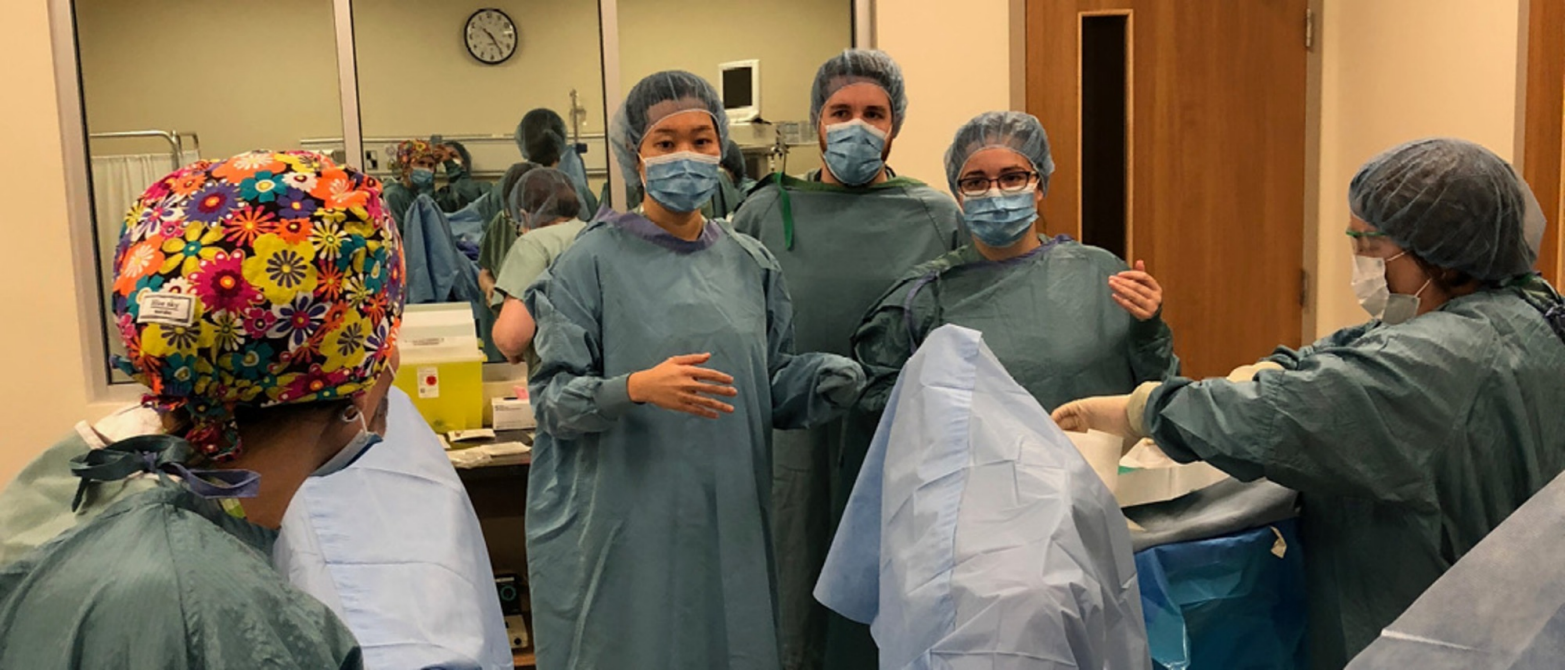
OB/GYN residents scrubbing in to participate in the sexual assault simulation scenario

The residents were briefed by the general surgeon upon arrival, scrubbed into the surgery and provided with an opportunity to examine the vaginal lacerations using the silicone model attached to the simulation mannequin (Figure 2).

Context

The Clinical Learning and Simulation Centre (CLSC) is a department within the Faculty of Medicine at MUN. The CLSC serves to train medical learners through the use of high-fidelity simulation and standardized patients, within a safe clinical environment to advance their surgical skills. Recently, the CLSC hosted a sexual assault simulation for OB/GYN residents, which consisted of a mannequin-based simulation within a simulated operating room, where residents were required to repair a vaginal laceration that was the result of a violent sexual assault.

Unrelated to the user-based product evaluation but relevant to the context of the scenario, the residents followed up the clinical simulation with an opportunity to rehearse their communication and empathy skills, which involved consoling a standardized patient who acted as the victim of the sexual assault. Standardized patients are actors who are recruited, trained, and scripted with a medical scenario in which they play the role of the patient. Medical trainees benefit from increased realism when speaking with real actors during simulation training as opposed to artificial mannequins.

**Figure 3:**
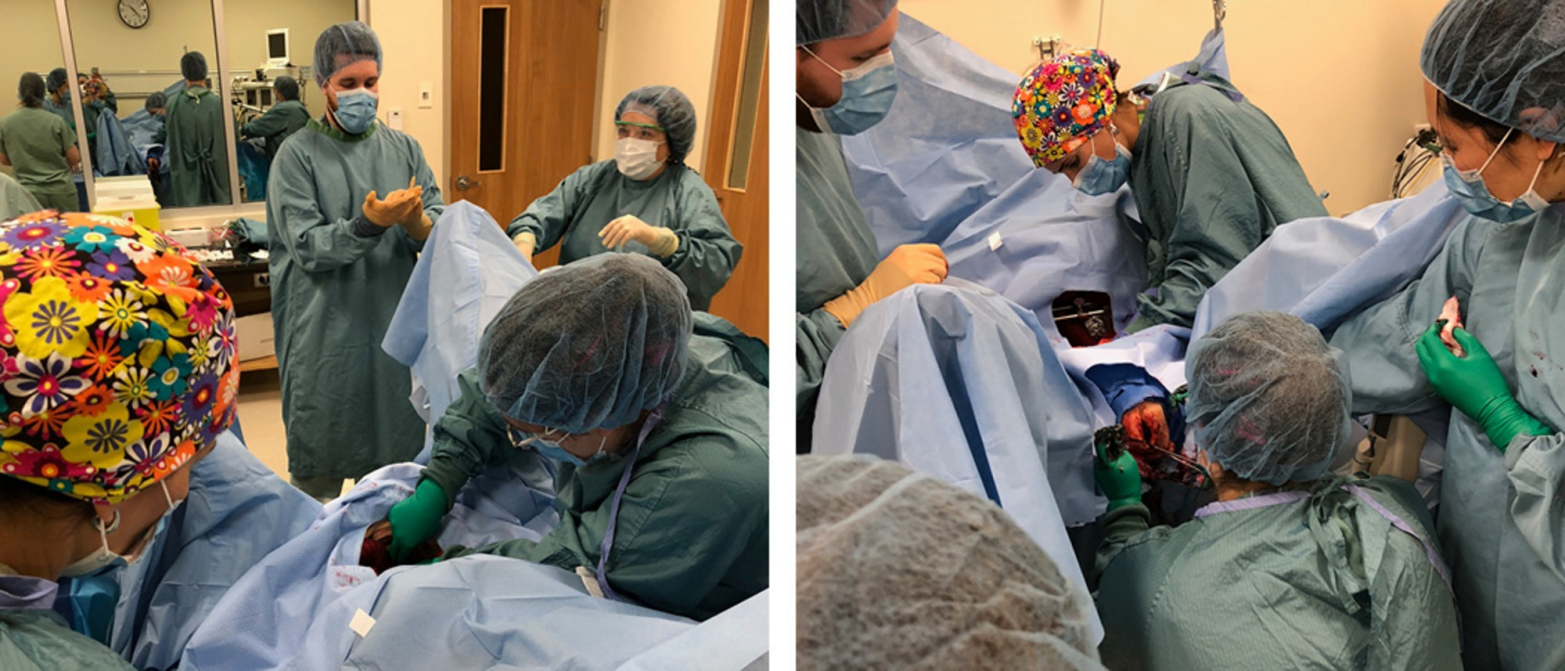
The rehearsal of laceration repair on the silicone model used in the sexual assault simulation

Following the procedure, the standardized patient acted as the sexual assault victim, whom the residents had to explain the procedure to as well as provide options to allow the use of a forensic evidence kit or to press criminal charges (Figure 3).

Input

**Figure 4:**
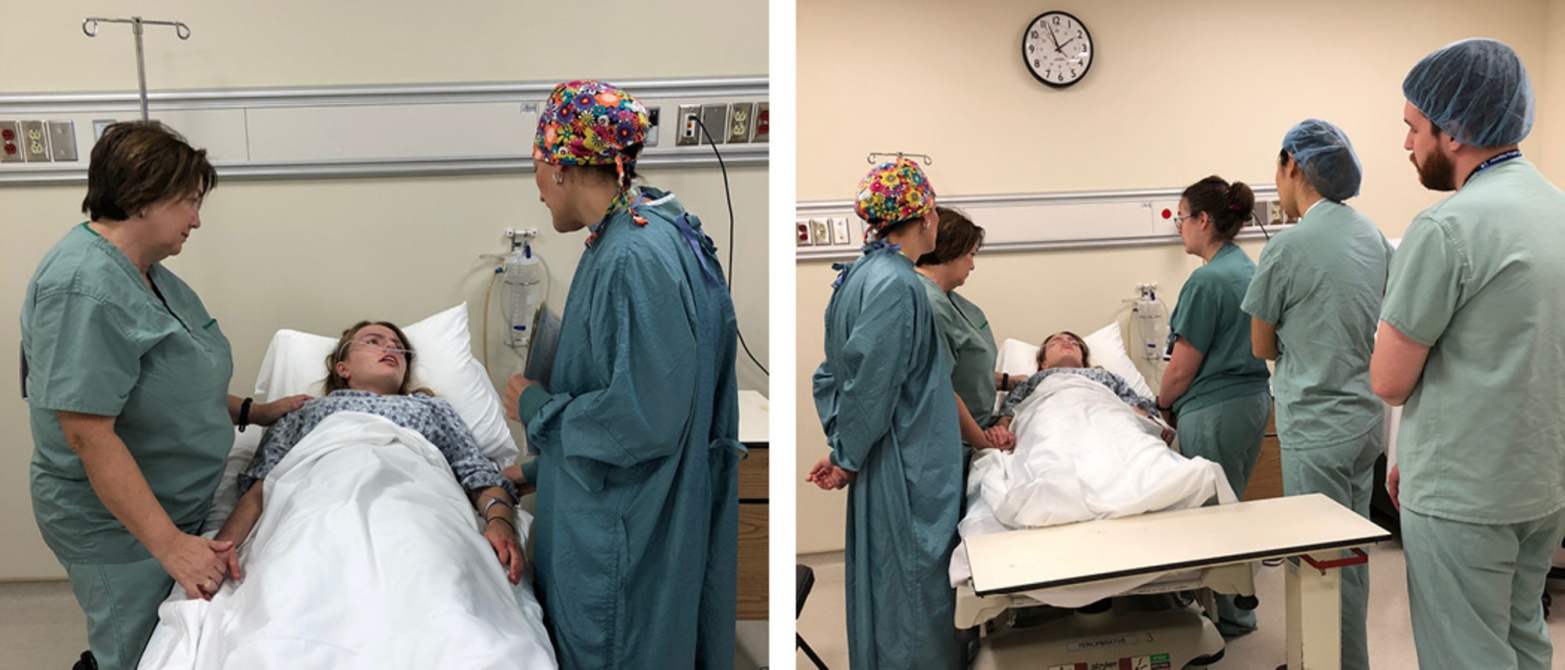
Standardized patient receiving information about her assault and surgery

The simulation scenario included a mannequin with the silicone vaginal model attached, which was covered with artificial blood for improved realism. The mannequin was placed on an operating room table and positioned with feet in stirrups, which accurately represented a patient about to be assessed, diagnosed, and treated for such a procedure under anesthetic (Figure 4).

The staff involved included two OB/GYN senior staff, who acted as the general surgeon and the nurse to allow the residents an opportunity to rehearse the vaginal repair. Also included in the scenario were two additional standardized confederates acting as the scrub nurse and anesthesiologist. In addition, a complete anesthetic setup, as well as surgical tools for the procedure, were provided prior to the scenario.

Process

For the sexual assault simulation scenario, residents entered the operating room expecting an urgent care case but did not know the details or severity. Upon their entrance into the operating room, the general surgeon updated the OB/GYN residents on the situation of the patient and what was to occur for the procedure. She informed them about the severity of the injuries and that it was likely the result of sexual assault. She requested that the residents choose which one of them would act as the leading OB/GYN surgeon to repair the injuries. The other two residents were to assist the leading OB/GYN surgeon by observing and providing tools when required. The selected lead-resident assessed the silicone model internally to determine the extent of the injuries. Once assessed, the resident requested to have sutures and toothed forceps to perform the laceration repair. The silicone model required approximately 10 sutures to close the laceration, which involved a soft tissue tear mediolaterally across the perineal plane as well as internally along the vaginal canal. Upon suturing completion, the resident did a final assessment to confirm that the repair visually appeared to be accurate.

When the laceration repair simulation ended, the residents left the operating room and met the general surgeon and attending nurse in the adjoining room with the standardized patient laying in a hospital bed. The simulated time-lapse was said to be approximately one-hour post-procedure. The residents were required to inform the standardized patient about her injuries, which were a result of the sexual assault. Residents were required to counsel the patient as she learned about the incident and the physical injuries that she had endured.

**Figure 5:**
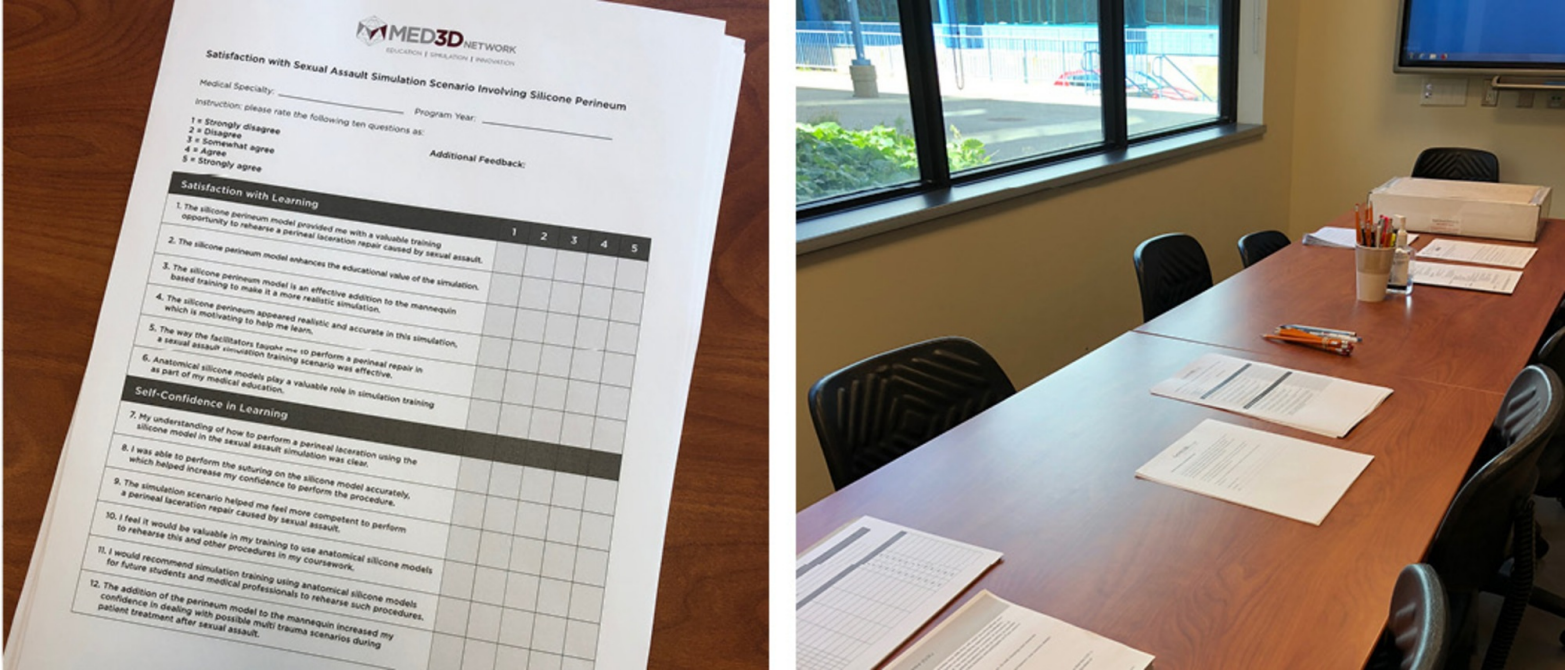
Product evaluation survey provided to OB/GY simulation resident participants

The simulation was repeated three times to allow 10 residents to participate. After each simulation, trainees were asked to complete a product evaluation survey that was provided in a separate room within the CLSC (Figure 5).

Products/Outcomes

**Figure 6:**
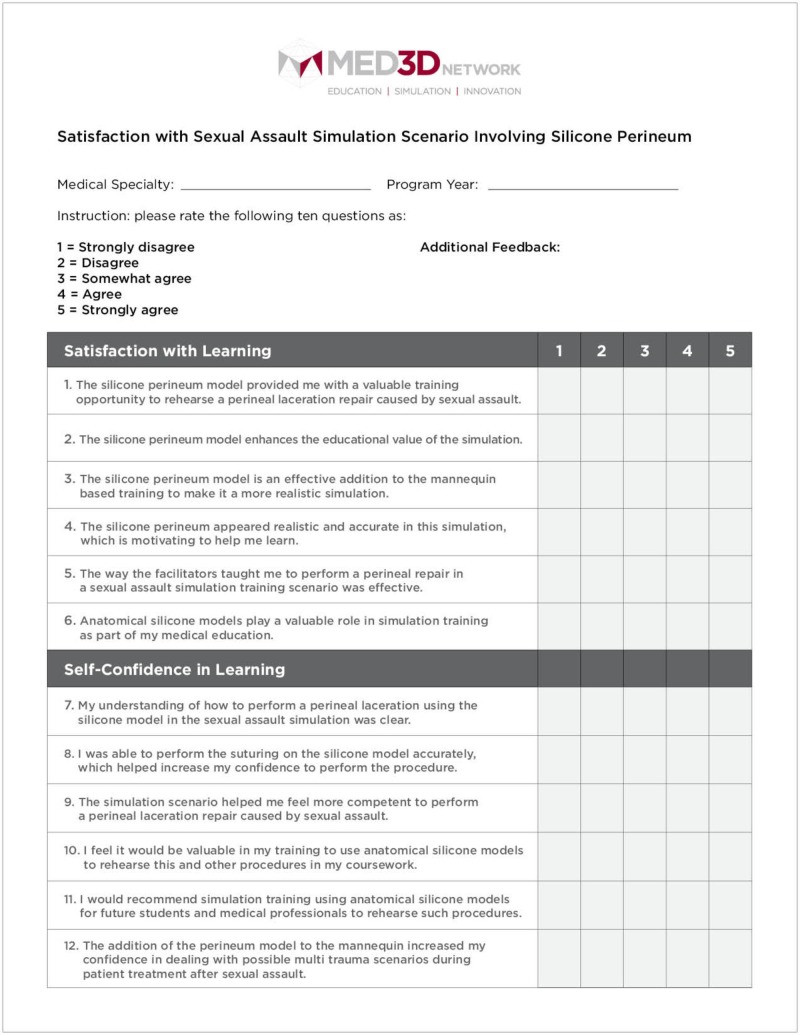
Sexual assault simulation scenario product evaluation survey

The user-based product evaluation survey was designed to assess the effectiveness of the silicone model used in conjunction with the simulation mannequin to improve the realism of the scenario by creating a hybrid training scenario. The survey provided residents with an opportunity to deliver their feedback on the use of the silicone model within the simulation scenario and how it could be improved to become a valuable addition to their ongoing simulation training (Figure 6).

**Figure 7:**
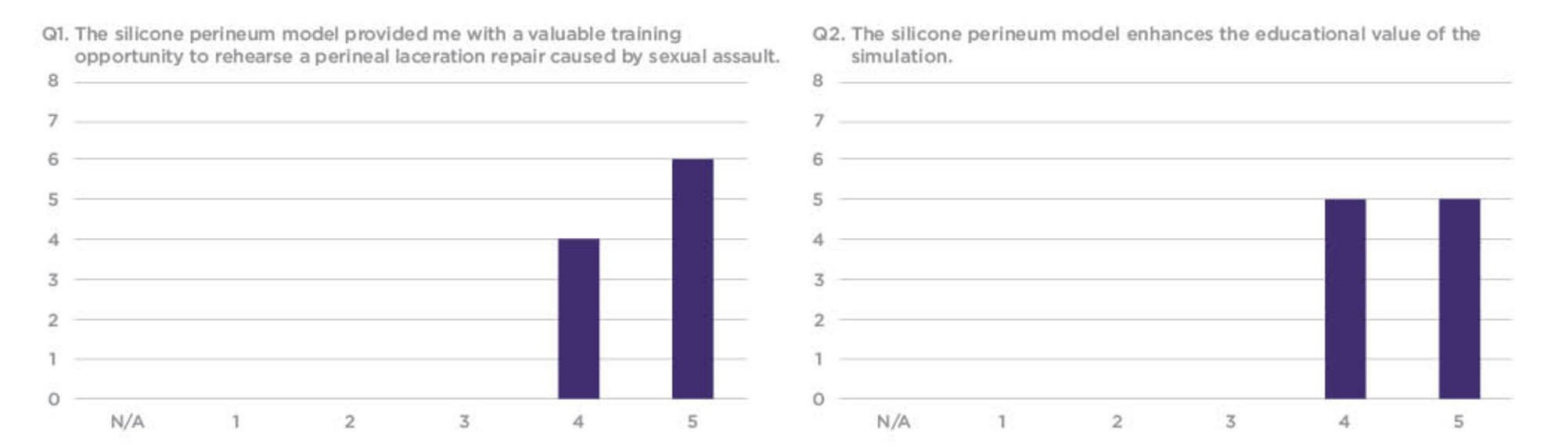
Q1 and Q2 results from the sexual assault simulation scenario participant product evaluation survey Y-axis: Frequency of responses; X-axis: Referring to the response anchors as related to each number respectively in Figure 6

**Figure 8:**
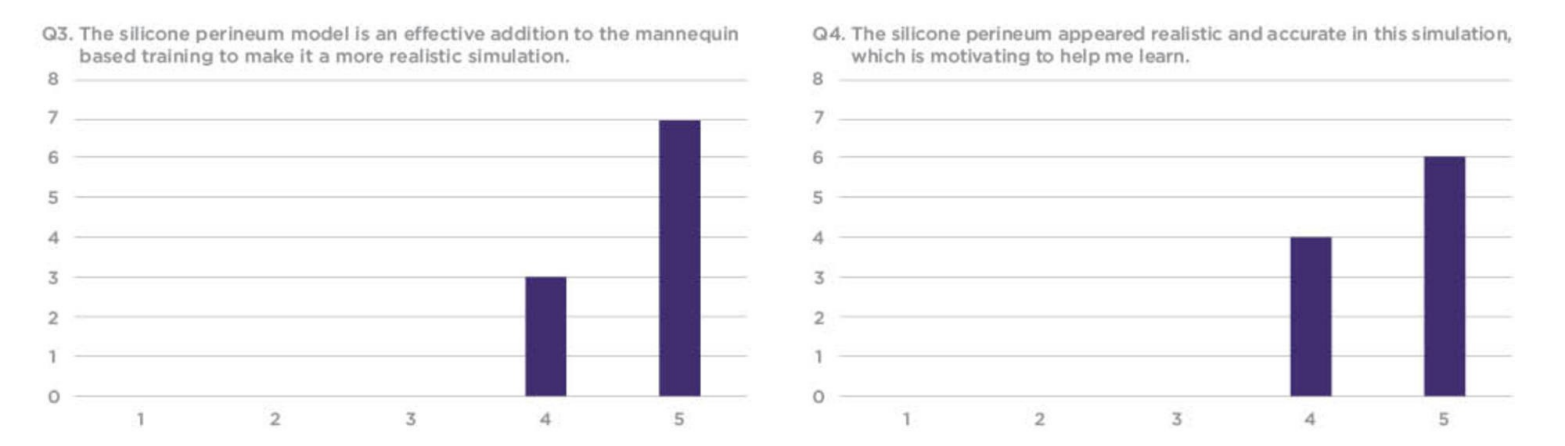
Q3 and Q4 results from the sexual assault simulation scenario participant product evaluation survey Y-axis: Frequency of responses; X-axis: Referring to the response anchors as related to each number respectively in Figure 6

**Figure 9:**
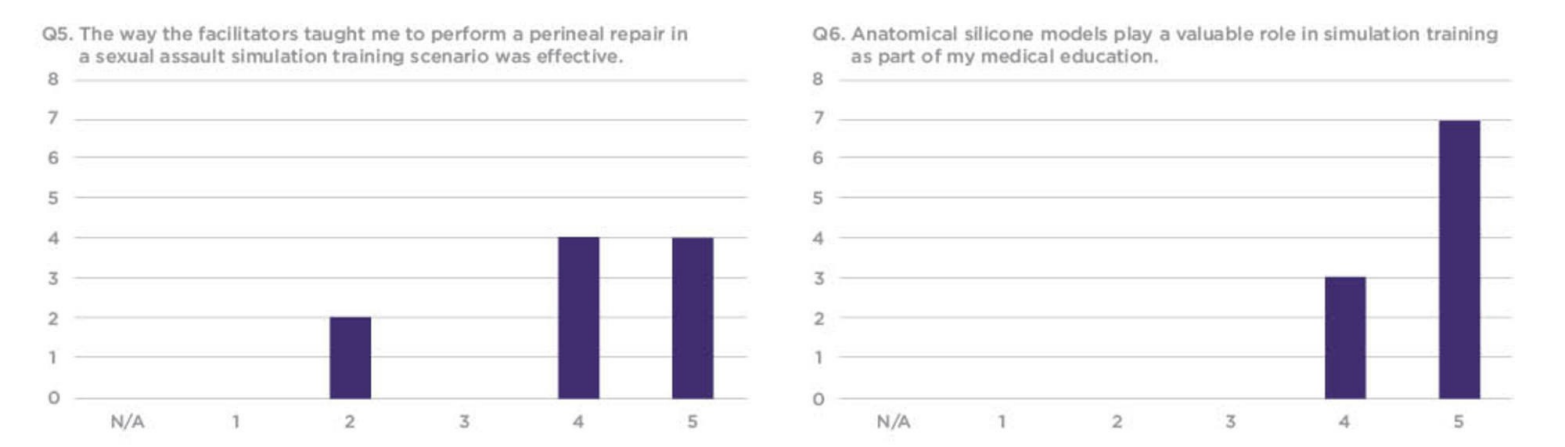
Q5 and Q6 results from the sexual assault simulation scenario participant product evaluation survey Y-axis: Frequency of responses; X-axis: Referring to the response anchors as related to each number respectively in Figure 6

**Figure 10:**
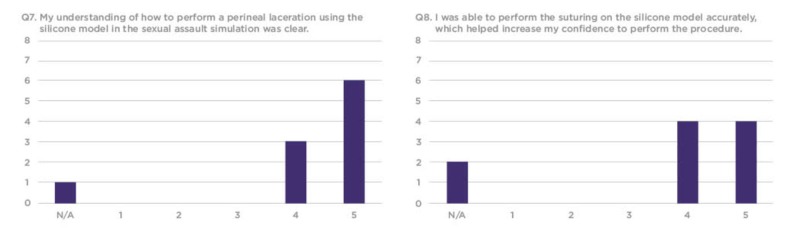
Q7 and Q8 results from the sexual assault simulation scenario participant product evaluation survey Y-axis: Frequency of responses; X-axis: Referring to the response anchors as related to each number respectively in Figure 6

**Figure 11:**
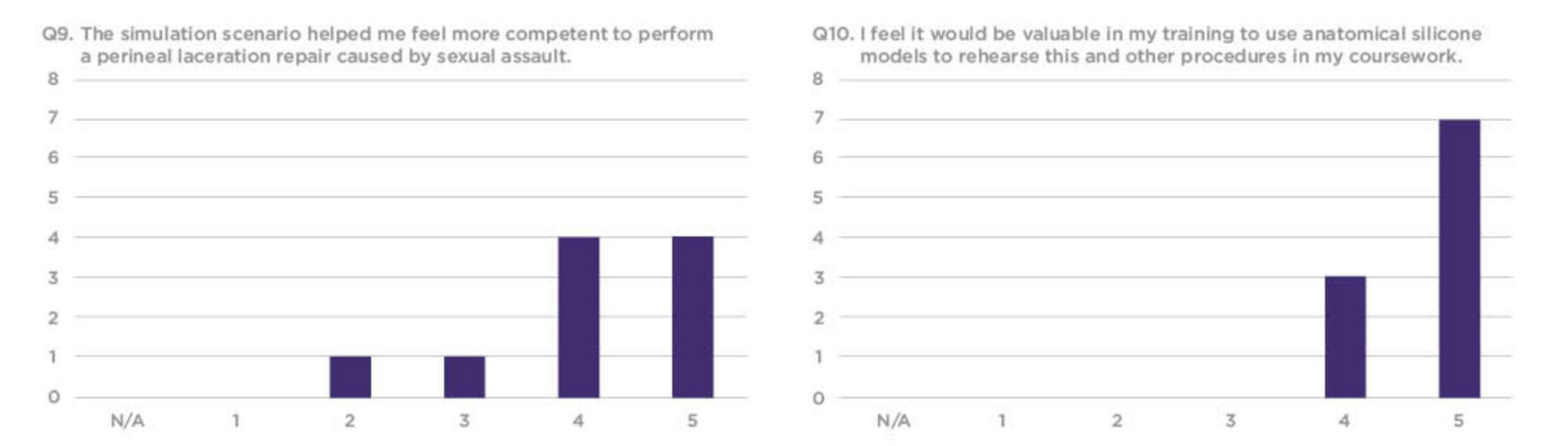
Q9 and Q10 results from the sexual assault simulation scenario participant research survey Y-axis: Frequency of responses; X-axis: Referring to the response anchors as related to each number respectively in Figure 6

**Figure 12:**
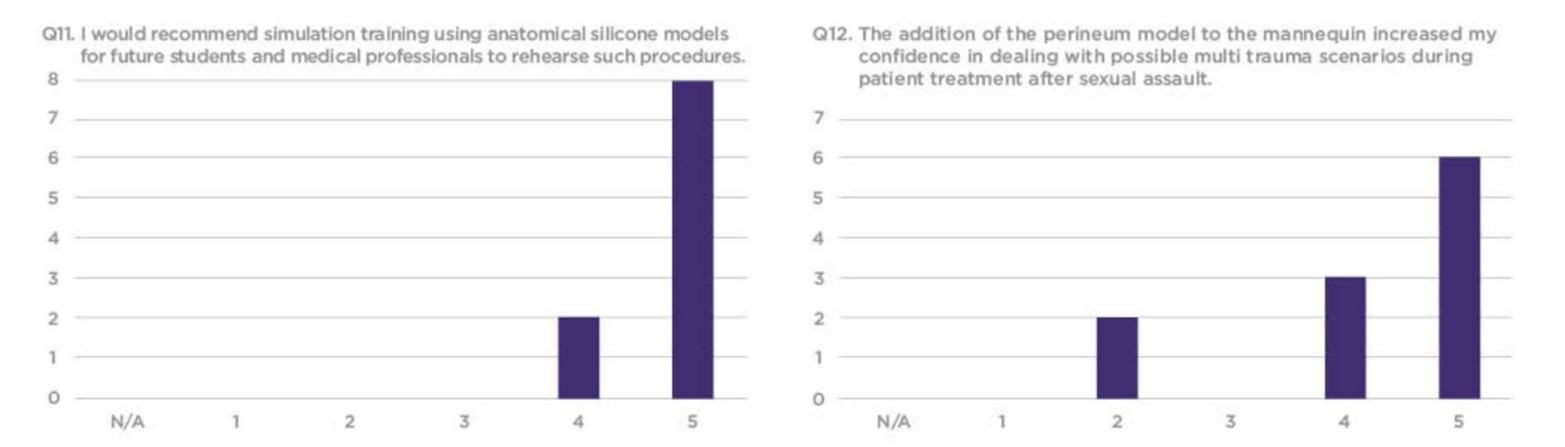
Q11 and Q12 results from the sexual assault simulation scenario participant research survey Y-axis: Frequency of responses; X-axis: Referring to the response anchors as related to each number respectively in Figure 6

The survey responses were compiled using a 1-5 Likert scale, requesting that trainees rate each question based on their personal experience. Overall, residents found the silicone vaginal model to be an effective and realistic tool with educational value to better prepare them for sexual assault cases in their future clinical practice (Figures 7-12).

The evaluation survey results revealed that a majority of the residents considered the simulation to be realistic and to be an effective teaching tool. Several residents, however, did not feel that the simulation increased their competency in repairing vaginal lacerations caused by sexual assault. This could be because the most experienced resident often repaired the laceration while more novice residents only assisted in the procedure (Figures 11-12).

## Discussion

SBME training provides residents with the opportunity to increase their clinical confidence and competency by allowing them to rehearse technical skills in a safe and controlled clinical environment. Sexual assault cases are particularly sensitive; therefore, residents could benefit from sexual assault simulation training to increase their confidence and competency pertaining to such cases. Most residents are not exposed to sexual assault cases throughout their training [14]. Simulation training could serve to fill this problematic gap in resident training using silicone models produced from economical 3D printed molds.

The silicone vaginal model used in the sexual assault simulation scenario was well received by the residents. Overall, residents felt that the silicone model provided them with a valuable training opportunity, which enhanced the educational component of the simulation session (survey questions 1 and 2). The survey results also reported positive responses in regard to the realism of the model and its effectiveness in combination with the mannequin (survey question 3). It was noted that the model can play a valuable role in the resident’s future educational learning, which they would also strongly recommend to other trainees and medical professionals (survey questions 6 and 11). Some of the lowest scored questions involved the educational direction about how to technically repair the injuries using the silicone model. Without proper instruction using the models prior to their use in a sexual assault simulation scenario, the residents felt that it did not significantly increase their competency or confidence to repair such injuries (survey questions 8 and 9).

Taking the lowest-ranked questions into consideration, a suggested improvement would be to introduce the residents to the technical use of the models prior to using them in more formal simulation scenarios, so the trainees become familiar with the haptic feedback of the silicone models in response to suturing. Since the models are a novel and newly accessible tool within the university medical education program at MUN, the use of the models is thought to become increasingly widespread in the near future, providing many new learners multiple opportunities to access the models for technical rehearsal before registering for the simulation training sessions. Another option would be to have the residents attend a brief 30-minute technical repair workshop, using the silicone models prior to beginning the sexual assault simulation scenario. Once complete, the residents could then apply their hands-on skills, learned in the technical workshop, within the simulation scenario.

Such task trainers would be beneficial to OB/GYN residency programs across Canada to help prepare their residents for clinical practice. Primarily, the task trainer would provide residents with an opportunity to refine their surgical skills in the context of vaginal lacerations as a result of sexual assault. Additionally, this task trainer could be used for simulation exercises similar to the one presented above. The task trainer used in conjunction with simulation would prepare residents to effectively manage victims of sexual assault. Such progressive learning paradigms, where component skills are practiced separately before the entire procedure is performed have been shown to be effective in both fundamental psychomotor skills acquisition and SBME [16-17].

## Conclusions

Within the context of this sexual assault simulation scenario, the silicone models developed were considered to be effective training tools, providing increased realism in combination with the mannequin for the rehearsal of vaginal laceration repair. The survey results revealed that the silicone models can potentially play a valuable role in the resident’s future educational learning, which they would also strongly recommend to other trainees and medical professionals. It was also noted that several residents did not feel that the simulation training increased their competency or confidence in repairing vaginal lacerations that were caused by sexual assault. This was most likely due to the lack of pre-training using the silicone models to better understand the haptic feedback of the material. In future sexual assault simulation training, it would also be worthwhile to investigate the benefits of providing each resident with a laceration repair rehearsal opportunity, as well as a technical learning workshop prior to using the silicone models within a sexual assault simulation scenario.
